# Variation in Oral Board Examination Accommodations Among Specialties

**DOI:** 10.1001/jamanetworkopen.2024.10127

**Published:** 2024-05-07

**Authors:** Dana G. Rowe, Antoinette J. Charles, Emily J. Luo, Alissa M. Arango, James E. Herndon, Harrison Hockenberry, Cynthia K. Shortell, C. Rory Goodwin, Melissa M. Erickson

**Affiliations:** 1 Duke University School of Medicine, Durham, North Carolina; 2 Department of Biostatistics and Bioinformatics, Duke University Medical Center, Durham, North Carolina; 3 Department of Neurosurgery, Duke University Medical Center, Durham, North Carolina; 4 Department of Surgery, Duke University Medical Center, Durham, North Carolina; 5 Department of Orthopedic Surgery, Duke University Medical Center, Durham, North Carolina

## Abstract

**Importance:**

Board certification can have broad implications for candidates’ career trajectories, and prior research has found sociodemographic disparities in pass rates. Barriers in the format and administration of the oral board examinations may disproportionately affect certain candidates.

**Objective:**

To characterize oral certifying examination policies and practices of the 16 Accreditation Council for Graduate Medical Education (ACGME)–accredited specialties that require oral examinations.

**Design, Setting, and Participants:**

This cross-sectional study was conducted from March 1 to April 15, 2023, using data on oral examination practices and policies (examination format, dates, and setting; lactation accommodations; and accommodations for military deployment, family emergency, or medical leave) as well as the gender composition of the specialties’ boards of directors obtained from websites, telephone calls and email correspondence with certifying specialists. The percentages of female residents and residents of racial and ethnic backgrounds who are historically underrepresented in medicine (URM) in each specialty as of December 31, 2021, were obtained from the Graduate Medical Education 2021 to 2022 report.

**Main Outcome and Measures:**

For each specialty, accommodation scores were measured by a modified objective scoring system (score range: 1-13, with higher scores indicating more accommodations). Poisson regression was used to assess the association between accommodation score and the diversity of residents in that specialty, as measured by the percentages of female and URM residents. Linear regression was used to assess whether gender diversity of a specialty’s board of directors was associated with accommodation scores.

**Results:**

Included in the analysis were 16 specialties with a total of 46 027 residents (26 533 males [57.6%]) and 233 members of boards of directors (152 males [65.2%]). The mean (SD) total accommodation score was 8.28 (3.79), and the median (IQR) score was 9.25 (5.00-12.00). No association was found between test accommodation score and the percentage of female or URM residents. However, for each 1-point increase in the test accommodation score, the relative risk that a resident was female was 1.05 (95% CI, 0.96-1.16), and the relative risk that an individual was a URM resident was 1.04 (95% CI, 1.00-1.07). An association was found between the percentage of female board members and the accommodation score: for each 10% increase in the percentage of board members who were female, the accommodation score increased by 1.20 points (95% CI, 0.23-2.16 points; *P* = .03).

**Conclusions and Relevance:**

This cross-sectional study found considerable variability in oral board examination accommodations among ACGME-accredited specialties, highlighting opportunities for improvement and standardization. Promoting diversity in leadership bodies may lead to greater accommodations for examinees in extenuating circumstances.

## Key Points

**Question:**

Is there variation by specialty in test accommodations for oral board examinations?

**Findings:**

In this cross-sectional study of policies and practices of all 16 Accreditation Council for Graduate Medical Education–accredited specialties that require oral certifying examinations, considerable variation in examination accommodation policies and practices was found. Specialties with more accommodating oral examination policies and practices tended to have more female and underrepresented in medicine residents as well as more females on their boards of directors.

**Meaning:**

Findings of this study suggest there is opportunity for improving and possibly standardizing test accommodations during the oral board certification examination process.

## Teaser

This cross-sectional study assesses variations in accommodations for oral certifying examinations among Accreditation Council for Graduate Medical Education–specialties.

## Introduction

Among US medical schools, certain aspects of the oral board certification process are standardized, including an eligibility period for taking the examination 5 to 8 years after completion of residency and requirements for earning a passing score on a written examination (ie, qualifying examination) before taking the oral examination (ie, certifying examination), paying fees, having current unrestricted licensure and hospital privileges, and meeting active practice requirements.^1^ However, despite these standardizations, there remains notable variation in certification practices by individual specialty boards.^1^ Whereas the written qualifying examination is largely a test of knowledge, oral certifying examinations serve to assess the candidate’s ability to describe appropriate patient management in case-based scenarios. These cases may be theoretical or based on a list of the candidate’s own cases, depending on the policies of each specialty board. Prior to the COVID-19 pandemic, the oral certifying examinations were largely held in person, and the setting in which they were held varied by specialty (hotel rooms, hotel suites, conference halls, or testing centers). The pandemic forced certifying agencies to adapt, and many certifying bodies temporarily transitioned to holding oral certifying examinations virtually.

The board certification process has broad implications for physicians’ academic promotion as well as compensation, with many institutions requiring job applicants to be board certified.^2^ Given these implications, as well as the variation in board certifying practices among specialties, it is important to ensure that the certifying process is equitable. Barriers in the format and administration of the oral board examinations have been previously described and may affect military recruits, nursing mothers, and other candidates who are disproportionately disadvantaged in terms of travel, cost, or other factors.^3,4^ In addition, recent studies have found differences in demographics of physician pass rates for oral board examinations, with candidates from minoritized racial and ethnic groups, as well as female candidates who are married with children at the time of internship being less likely to pass certifying examinations on their first attempt.^5^ These findings highlight potential barriers to passing oral board examinations that may contribute to well-described racial-, ethnic-, and gender-based disparities in medical specialties.^6,7^ In addition to well-documented challenges in achieving gender parity among the specialties, studies have found that physicians who are female, Black or African American (hereafter, Black), or Hispanic experience higher attrition rates in graduate medical programs.^5,7,8,9,10,11^ It is thus important to identify and address any barriers so as to make the board certification process more equitable. Therefore, this study sought to characterize the landscape of US oral certifying examinations by identifying the accommodation policies and practices of specialties requiring these examinations.

## Methods

### Study Design

The institutional review board of Duke University deemed this study exempt from review and waived the informed content requirement because only publicly available data were used. This cross-sectional study followed the Strengthening the Reporting of Observational Studies in Epidemiology (STROBE) reporting guideline .

### Test Accommodation Scoring

All 16 Accreditation Council for Graduate Medical Education (ACGME)–accredited specialties requiring oral board examinations were included in the study: anesthesia, colon and rectal surgery, emergency medicine, interventional radiology, neurological surgery, obstetrics and gynecology (OBGYN), ophthalmology, orthopedic surgery, otolaryngology, physical medicine and rehabilitation, plastic surgery, radiation oncology, general surgery, thoracic and cardiac surgery, urology, and vascular surgery. Each specialty board’s website was accessed in March 2023 to assess for the following policies surrounding their oral examinations: format (in-person or virtual), number of test dates offered (1, 2, or >2 days), test setting (hotel suite, conference center, testing center, or virtual), lactation accommodations available (lactation rooms or areas or extra time offered for nursing), and accommodations for military deployment, family emergency, or medical leave. The policy data were recorded, and each specialty board was then contacted via telephone to verify the data acquired from the board websites. A standardized script (eMethods in Supplement 1) was followed with questions assessing each specialty board’s policies and practices surrounding the oral certifying examination. These telephone calls served to verify the information collected from the board website and provided boards with the opportunity to elaborate on their oral examination policies and practices. All 16 specialty boards were successfully contacted.

Each accommodation policy was scored on a scale as described in the policy scoring key (Table 1), with higher total scores indicating more accommodations offered to candidates. Two independent observers (E.J.L. and A.M.A.) applied the scoring key to the policies, and discrepancies were resolved by a third reviewer (D.G.R.). This scoring system was adapted from Bristol et al^12^ and Welch et al^13^ to be relevant to oral certifying examinations. Subtotals were tallied for oral certifying examination policies and practices. Scores for each policy ranged from 0 to 2. A total possible test accommodation score ranging from 0 to 13 was calculated for each specialty based on the degree of accommodation offered by their policies and practices surrounding the oral certifying examination.

**Table 1.** Oral Certifying Examination Policies and Practices Scoring Key

Policy or Practice	Score	Scoring definition
Examination format	0	In-person
	1	Virtual
No. of examination dates offered	0	1
	1	2
	2	>2
Examination location	0	Hotel suites
	1	Conference center
	2	Testing center or virtual
Accommodations for personal medical leave	0	No alternate date or virtual examination offered; if unavailable, candidate must postpone examination by 1 y
	1	No specific policy but state they may be able to accommodate changes
	2	Virtual alternative offered (if format is in-person) or candidate may switch to another date during that same year
Accommodations for family emergency	0	No alternate date or virtual examination offered; if unavailable, candidate must postpone examination by 1 y
	1	No specific policy but state they may be able to accommodate changes
	2	Virtual alternative offered (if format is in-person) or candidate may switch to another date during that same year
Accommodations for military deployment	0	No alternate date or virtual examination offered; if unavailable, candidate must postpone examination by 1 y
	1	No specific policy but state they may be able to accommodate changes
	2	Virtual alternative offered (if format is in-person) or candidate may switch to another date during that same year
Lactation accommodations	0	No rooms available or not offered
	1	In-person examination offering extra break time but no lactation rooms or spaces available or virtual examination with no extra time or accommodations for lactation
	2	Lactation rooms or spaces available or virtual examination with extra time or accommodations for lactation

### Demographics of Boards of Directors and Residents

The websites of all ACGME-accredited specialties requiring oral board examinations were reviewed to determine the gender makeup of the board of directors or board of trustees. Each member listed online as part of the board as of March 25, 2023, was included toward the total count of board members. Each board member’s gender was determined based on the pronouns used in the descriptive biographies on the board website. When no biography was available, the board member’s preferred pronouns were determined through an internet search to identify other biographies that specified the individual’s preferred pronouns. Each specialty board was then contacted via telephone to confirm the number of individuals on their board of directors and the gender makeup of their board. When we were unable to reach the board via telephone, a follow-up email was sent to confirm the gender makeup of the board. This process was adapted from Saxena et al.^14^ All 16 specialty boards were able to confirm the gender makeup of their board of directors.

The percentage of female residents and residents of racial and ethnic groups who have been historically underrepresented in medicine (URM) were documented for each specialty using the Graduate Medical Education 2021 to 2022 report.^6^ Residents identifying as American Indian or Alaska Native, Black, Native Hawaiian or Other Pacific Islander, or of Hispanic ethnicity were classified as URM residents, according to the American Association of Medical Colleges’ definition of underrepresented minorities in medicine.^15^

### Statistical Analysis

Descriptive statistics, including means (SDs) and medians (IQRs), were calculated to summarize accommodation scores. A Poisson regression model with an offset adjusting for overdispersion was used to examine the association between the accommodation score and the percentages of female residents and URM residents. The offset was defined as the logarithm of the total number of residents. For each point unit increase in the accommodation score, the relative risk that a resident was female or a URM resident was computed. The analysis aimed to test the hypothesis that higher accommodation scores were associated with increased resident diversity in a specialty. Additionally, linear regression was used to evaluate the association between the percentage of female board members and the accommodation score, testing the hypothesis that greater female representation on a board would be associated with higher levels of accommodation. A *P* < .05 was set as the threshold for statistical significance. Analyses were generated using SAS, version 9.4 (SAS Institute Inc) and RStudio, version 2023.06 (Posit PBC).

## Results

Included in the analysis were 16 US medical specialties that required oral board examinations as of April 15, 2023, with a total of 46 027 residents (19 494 females [42.4%] and 26 533 males [57.6%]). Of these individuals, 6983 (15.2%) were URM residents and 39 044 (84.8%) were White residents. The boards of the specialty organizations included 233 members (81 females [34.8%] and 152 males [65.2%]).

### Accommodations Scores and Individual Board Policies

Accommodation scores were assigned for each of the 16 specialties that require oral board examinations, with a mean (SD) total accommodation score of 8.28 (3.79) and a median (IQR) score of 9.25 (5.00-12.00) (Table 1). Emergency medicine had the highest total accommodation score (13). Four specialties (25.0%) had a score of 12 (anesthesia, interventional radiology, OBGYN, and radiation oncology). The 3 specialties with the lowest accommodation scores were colon and rectal surgery (score of 1), urology (score of 4), and thoracic and cardiac surgery (score of 4). Accommodation scores for each rating category, as well as total scores by specialty, are presented in Table 2 and the eFigure in Supplement 1. Descriptions of each specialty board’s accommodation policies can be found in the eAppendix in Supplement 1.

**Table 2.** Accommodation Scores by Specialty

	Scores for examination characteristics			Scores for accommodations available				Total accommodation score
	Format^a^	Dates offered	Location	Medical leave	Family emergency	Military deployment	Lactation	
Specialty								
Anesthesia	0	2	2	2	2	2	2	12
Colon and rectal surgery	0	0	0	0	0	0	1	1
Emergency medicine	1	2	2	2	2	2	2	13
Interventional radiology	1	1	2	2	2	2	2	12
Neurological surgery	1	1	2	2	2	2	1	11
Obstetrics and gynecology	0	2	2	2	2	2	2	12
Ophthalmology	1	1	2	1	1	1	2	9
Orthopedic surgery	0	1	1.5^b^	2	2	2	1^b^	9.5
Otolaryngology	1	0	2	0	0	1	1	5
Physical medicine and rehabilitation	1	0	2	0	0	1	2	6
Plastic surgery	0	0	0	2	2	2	0	6
Radiation oncology	1	1	2	2	2	2	2	12
General surgery general	1	1	2	2	2	2	1	11
Thoracic and cardiac surgery	0	0	2	0	0	0	2	4
Urology	0	0	2	0	0	0	2	4
Vascular surgery	1	0	2	0	0	0	2	5
Score								
Mean (SD)	0.56 (0.51)	0.75 (0.77)	1.72 (0.68)	1.19 (0.98)	1.19 (0.98)	1.31 (0.87)	1.56 (0.63)	8.28 (3.79)
Median (IQR)	1.00 (0.00-1.00)	1.00 (0.00-1.00)	2.00 (2.00-2.00)	2.00 (0.00-2.00)	2.00 (0.00-2.00)	2.00 (0.75-2.00)	2.00 (1.00-2.00)	9.25 (5.00-12.00)

^a^ Maximum score for examination format was 1, whereas maximum score for all other categories was 2.

^b^ The oral board examinations for orthopedic surgery are traditionally held in an exhibit space in a hotel. The specialty board added a second date for oral examinations that will be held at a testing center (see the eAppendix in Supplement 1 for details). The score of 1.5 is an average of the examination location scores for these 2 separate testing sites. Similarly, the policies for lactation represent an average of the lactation accommodations afforded at both testing sites.

### Oral Board Examination Administration

The mean (SD) accommodation scores across the specialties for areas related to examination administration were 0.56 (0.51) for test format (in person or virtual), 0.75 (0.77) for number of examination dates, and 1.72 (0.68) for locations offered (hotel, conference center, testing center or virtual) (Table 2). Nine of 16 specialties (56.2%) offered a virtual examination (emergency medicine, interventional radiology, neurological surgery, ophthalmology, otolaryngology, physical medicine and rehabilitation, radiation oncology, general surgery, and vascular surgery). Three specialties (18.8%) offered more than 2 examination dates (anesthesia, emergency medicine, and OBGYN). Seven specialties (43.8%) offered only 1 examination date (colon and rectal surgery, otolaryngology, physical medicine and rehabilitation, plastic surgery, thoracic and cardiac surgery, urology, and vascular surgery). Two specialties (plastic surgery and colon and rectal surgery) hosted their oral certifying examinations in hotel suites rather than a testing center, conference center, or virtually.

### Medical Leave Policies

Accommodations for medical leave were scored by whether candidates could switch their examination date or whether a virtual alternative was offered in place of an in-person examination. The mean (SD) medical leave accommodation score was 1.19 (0.98). Nine of 16 specialties (56.2%) had the highest level of test accommodations for medical leave (score of 2), offering at least 1 of these options. Six specialties (37.5%) had the lowest accommodation score for medical leave (score of 0), offering no accommodations for medical leave and requiring candidates unable to participate on the offered date to postpone their examinations by 1 year. Ophthalmology had a medical leave accommodation score of 1, as their policy requires candidates unable to participate on the offered date to cancel and reschedule for the next offered examination, although candidates may submit a letter requesting accommodations for review and approval by board leadership.

### Family Emergency and Military Deployment Policies

Family emergency leave policies and accommodation score were identical to those for medical leave across the 16 specialties. Nine specialties had family leave test accommodation scores of 2 (anesthesia, emergency medicine, interventional radiology, neurological surgery, OBGYN, orthopedic surgery, plastic surgery, radiation oncology, and general surgery). Six specialties had no accommodations for family leave (colon and rectal surgery, otolaryngology, physical medicine and rehabilitation, thoracic and cardiac surgery, urology, and vascular surgery). One specialty (ophthalmology) required candidates unable to participate on the offered date to cancel and reschedule their examination (but allowed the candidate to submit a special request to board leadership).

Specialties were generally more accommodating for leave associated with military deployment, with a total mean (SD) accommodation score of 1.31 (0.87). The 9 highest-scoring specialties for medical and family leave policies were equally accommodating for military deployment (Table 2).

### Lactation Policies

Specialties were the most accommodating regarding lactation policies, with a mean (SD) accommodation score of 1.56 (0.63). As shown in Table 2, 10 specialties offered the highest level of accommodation (score of 2), either offering a lactation room or space for in-person examinations or offering extra time or accommodations for virtual examinations. Five specialties had a score of 1 (colon and rectal surgery, neurological surgery, orthopedic surgery, otolaryngology, and general surgery) and offered either extra break time without a designated lactation room for in-person examination or a virtual examination with no additional time allocated.

### Representation of Female and URM Residents

**Figure 1.**
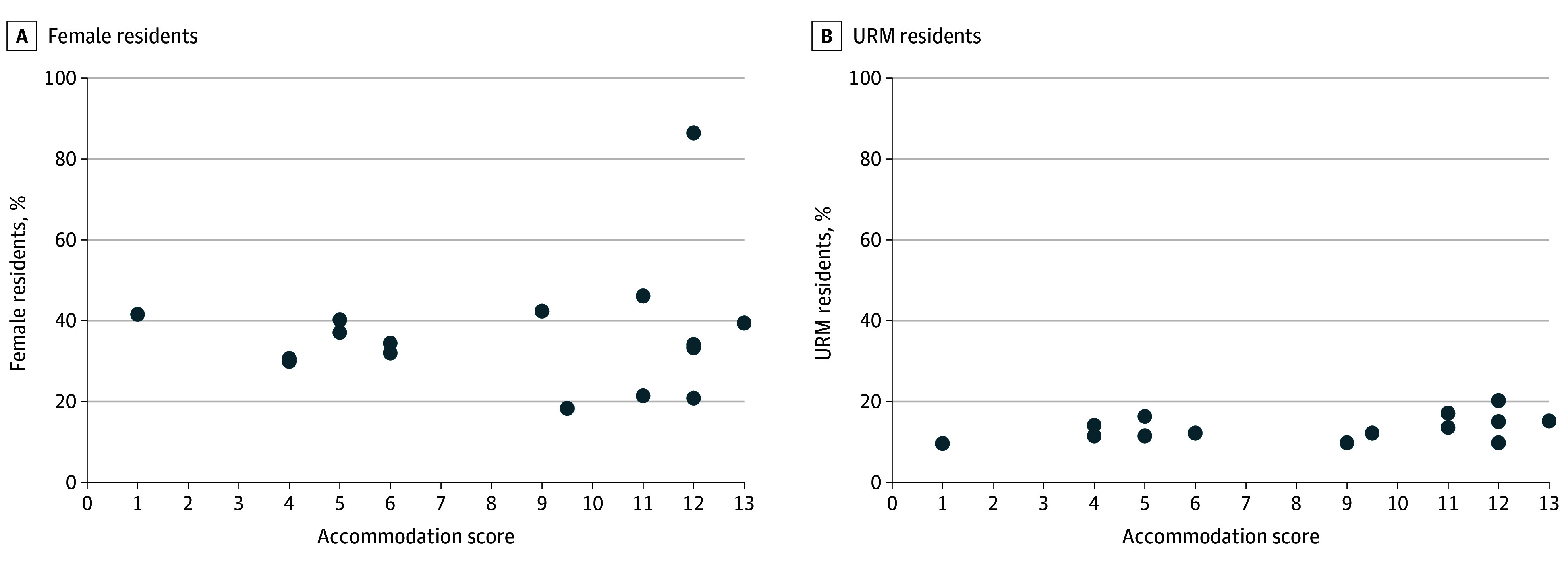
Association Between Accommodation Score and Percentage of Female Residents and Underrepresented in Medicine (URM) Residents

In analyzing the percentage of female residents in each specialty, OBGYN had the overwhelmingly highest percentage of female residents at 86.4% (Table 3). Orthopedic surgery had the lowest percentage of female residents at 18.3%. For all specialties other than OBGYN, fewer than 50.0% of residents were female. There was no association between accommodation score and percentage of female residents (Figure 1A): for each 1-point increase in the accommodation score, the relative risk that a resident was female was 1.05 (95% CI, 0.96-1.16).

**Table 3.** Residency Programs and Boards of Directors Demographics

Specialty	Total No. of residents^a^	No. (%)^a^		Total board members, No.	Female board members, No. (%)
		Female residents	URM residents^b^		
Anesthesia	6608	2255 (34.1)	991 (15.0)	14	5 (35.7)
Colon and rectal surgery	94	39 (41.5)	9 (9.6)	16	4 (25.0)
Emergency medicine	8659	3410 (39.4)	1316 (15.2)	17	8 (47.1)
Interventional radiology^c^	853	177 (20.8)	82 (9.6)	3	2 (66.7)
Neurological surgery	1563	334 (21.4)	212 (13.6)	15	2 (13.3)
Obstetrics and gynecology	5738	4959 (86.4)	1158 (20.2)	13	9 (69.2)
Ophthalmology	1433	606 (42.3)	140 (9.8)	18	9 (50.0)
Orthopedic surgery	4358	797 (18.3)	532 (12.2)	21	3 (14.3)
Otolaryngology	1741	700 (40.2)	200 (11.5)	18	6 (33.3)
Physical medicine and rehabilitation	1484	511 (34.4)	187 (12.6)	15	6 (40.0)
Plastic surgery	181	58 (32.0)	22 (12.2)	21	6 (28.6)
Radiation oncology	753	251 (33.3)	74 (9.8)	4	2 (50.0)
General surgery	9900	4561 (46.1)	1689 (17.1)	12	7 (58.3)
Thoracic and cardiac surgery^d^	504	151 (29.9)	58 (11.5)	20	5 (25.0)
Urology	1778	544 (30.6)	251 (14.1)	14	4 (28.6)
Vascular surgery	380	141 (37.1)	62 (16.3)	12	3 (25.0)

Abbreviation: URM, underrepresented in medicine.

^a^ Data acquired from Graduate Medical Education 2021 to 2022 (GME) report.^6^

^b^ Residents identifying as American Indian or Alaska Native, Black, Native Hawaiian or Other Pacific Islander, or of Hispanic ethnicity were classified as URM residents, according to the American Association of Medical Colleges’ definition of underrepresented minorities in medicine.

^c^ Includes interventional radiology–independent and interventional radiology–integrated in the GME report.

^d^ Includes thoracic surgery and thoracic surgery–integrated in the GME report.

Among the specialties, OBGYN and general surgery had the highest percentages of URM residents at 20.2% and 17.1%, respectively. No association was found between accommodation score and the percentage of URM residents (Figure 1B); however, for each unit increase in the accommodation score, the relative risk that a resident was a URM resident was 1.04 (95% CI, 1.00-1.07).

### Female Representation Among Board Members

**Figure 2.**
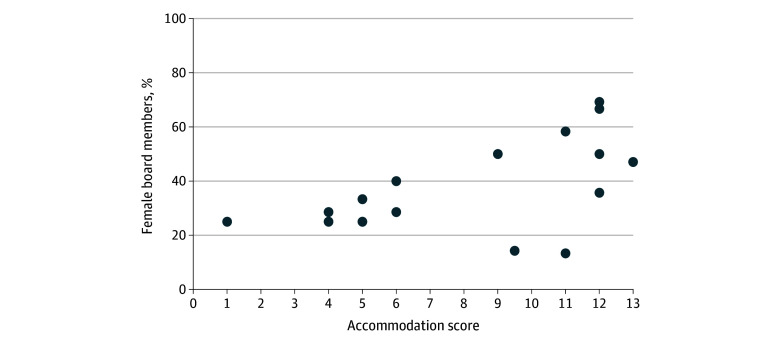
Association Between Percentage of Female Board Members and Accommodation Score

Regarding the percentage of female board members, OBGYN and interventional radiology had the highest percentage of female board members at 69.2% and 66.7%, respectively. There was an association between the percentage of female board members and accommodation score: for each 10% increase in female board membership, the accommodation score increased by 1.20 points (95% CI, 0.23-2.16 points; *P* = .03) (Figure 2).

## Discussion

Physician board certification is a standardized process that assesses the physician’s mastery of knowledge and skills obtained during residency. The policies and practices surrounding oral certifying examinations, including format, scheduling, location, and lactation policies, exhibit considerable variability, which may adversely affect certain groups and a candidates’ ability to become board certified. As this study shows, 56.2% (9 of 16) of specialties administer oral board examinations virtually. Of the specialties holding in-person oral examinations, there is variation in examination location, with some specialties using testing centers and others using conference centers or hotel suites. A prior perspective report has commented on the uncomfortable and potentially triggering nature of hotel-based examination settings.^3^ During the COVID-19 pandemic, many oral board examinations transitioned from being held in person to being held virtually, with greater flexibility in canceling and rescheduling examinations. At the time of this study, numerous specialties have returned to in-person examinations, yet some specialties remain completely virtual, noting increased flexibility of this examination format.^16,17,18^ Specialty boards should continue to reflect on the format in which their oral certifying examination is administered and consider whether their current format has the candidate’s best interests in mind.

Oral certifying examinations are a crucial step in the process of board certification for physicians. Unfortunately, circumstances may arise that necessitate accommodations for taking or rescheduling these examinations. Medical emergencies or family emergencies (eg, family member death) may prevent candidates from taking the oral board examinations. In addition, individuals in their third trimester of pregnancy may be under travel restrictions that prevent them from attending in-person oral board examinations.^19^ Nursing mothers may require the use of lactation rooms or extended break time to pump breast milk. Military deployment may require candidates to reschedule their examination or request accommodations, such as remote proctoring or alternative testing locations.^20^ The findings of this study show that there is notable variability in the oral qualifying board examination accommodations offered by each specialty board in the face of the aforementioned life events. Nonetheless, a lack of accommodation poses potential barriers to successful completion of the oral board examination, and can in certain cases (eg, pregnancy or lactation) disproportionately affect certain candidates. Given these implications, all ACGME-accredited specialties requiring oral board examinations should strive to have clear policies and procedures in place to accommodate these situations and to ensure fairness and equity in the board certification process.

The findings of this study suggest that there is room for standardization and increased flexibility among specialties concerning accommodation policies for certifying examinations. The boards of directors of ACGME-accredited specialties should reflect on their current policies and procedures and determine whether there are areas for improvement or increased accommodation. Educational researchers have shown that providing test accommodations to individuals who require them can lead to improved test scores for those individuals without affecting the average scores of those who do not require accommodations.^21^ This finding addresses concerns regarding the potential for accommodations to create an unfair advantage. Thus, standardizing accommodation policies across specialties could help ensure consistency in the board certification process while also accommodating the unique needs of individual candidates.

This study’s findings suggest a possible positive correlation between diversity and the degree of accommodation offered for oral board examinations. There was a direct association between the accommodation score and the percentage of female board members. As most members of most specialty boards of directors are male, a higher percentage of female board members may reflect diversity of thought and thus lends itself to more accommodating policies and practices surrounding oral board examinations. Furthermore, while the association between accommodation scores and percentage of URM residents was not statistically significant, the data indicate a trend by which specialties with higher accommodation scores have a greater percentage of URM residents. These findings are in line with research showing the association between workforce diversity and the adoption of flexible workplace practices.^22^ Diversity in leadership is also associated with increased innovation, greater depth and breadth of experience, and perspectives that improve the problem-solving and decision-making abilities of the organization.^23,24,25^ These factors highlight the importance of promoting diversity and inclusion of leaders within the committees and boards of directors of specialty accrediting bodies to inform the development of policies and practices for oral board examinations, as it may lead to greater flexibility and accommodation for examinees.

### Limitations

Several limitations need to be considered when interpreting the findings of this study. First, the study relied on publicly available data, which may be incomplete or outdated and could lead to discrepancies and inaccuracies. Nonetheless, the collected data were verified based on the input provided by a certifying specialist at each accrediting body. Furthermore, the policy scoring system used in this study is a limited method for evaluating the accommodations of a specialty’s oral board examination policies and practices. We developed this system based on work by Bristol et al^12^ and Welch et al.^13^ It is not a validated tool for assessing the accommodation level of oral board examinations. Moreover, the scoring system weights the different types of policy equally and thus does not account for differences in the importance and impact of each policy and practice evaluated. Values attributes to each policy and practice were intended to assess the degree of accommodations offered by each accrediting body; they are not meant to serve as a ranking system. Finally, using the percentage of boards of directors who are female or URM individuals as a proxy for diversity may not fully capture the range and intersectionality of factors that can contribute to potential marginalization, such as race, ethnicity, gender, socioeconomic background, and disability.

## Conclusions

In this study, there was considerable variation in the policies and practices of ACGME-accredited specialty boards surrounding oral board examinations, with notable opportunity for improvement and possible standardization of the oral board certification process. This cross-sectional study revealed that specialty boards with more accommodating oral board examination policies and practices tended to have more gender, racial, and ethnic diversity in their residency classes. Greater gender diversity of the boards of directors was associated with higher test accommodation scores. Promoting diversity in leadership bodies may lead to greater accommodations for examinees in extenuating circumstances.

## Notes

Supplement 1.
eMethods.
**eFigure.** Total Accommodation Score by Specialty **eAppendix.** Policies and Practices of Oral Board Examinations

Supplement 2.
Data Sharing Statement
