# Supplementary material for: Variation in Oral Board Examination Accommodations Among Specialties
Source: JAMA Netw Open. 2024 May 7;7(5):e2410127. doi: 10.1001/jamanetworkopen.2024.10127 (PMC11077388; doi:10.1001/jamanetworkopen.2024.10127)
Supplement: Supplement 1. — eMethods. eFigure. Total Accommodation Score by Specialty eAppendix. Policies and Practices of Oral Board Examinations [file jamanetwopen-e2410127-s001.pdf]

Supplemental Online Content

Rowe DG, Charles AJ, Luo EJ, et al. Variation in oral board examination accommodations among specialties. *JAMA Netw Open.* 2024;7(5):e2410127. doi:10.1001/jamanetworkopen.2024.10127

eMethods

eFigure. Total Accommodation Score by Specialty

eAppendix. Policies and Practices of Oral Board Examinations

This supplemental material has been provided by the authors to give readers additional information about their work.

eMethods

Script for Phone Calls with Certifying Organizations

<u>Goal</u>: To assess executive director/board administration's ability to provide information regarding accommodations and testing standards for oral examinations.

<u>Script:</u>

Introduction: "Hi, my name is [____]. I'm a medical student interested in [____] specialty. I was reviewing your website to learn about the path to becoming a board-certified [____] and wanted to see if I could get some more information about the oral exam?"

Script:

1. It appears that you offer these exams [____] times per year, [in person/virtually]. Is this correct?
    a. [Use information from spreadsheet to fill-in blanks].
2. *If in person* (if virtual, skip this question):
    a. Do all the examinees come to the same location or are the exams at a testing center?
    b. What is the setting of the examinations (e.g., conference room, pods, hotel rooms, etc.)?
3. Do you have a policy for alternate dates for medical emergencies (Y/N)?
    a. *If in-person*: Do you offer a virtual option in the event of medical emergencies? (Y/N)
4. Do you have a policy for alternate dates for family emergencies (e.g., death of a family member)? (Y/N)
    a. *If in-person*: Do you offer a virtual option in the event of family emergencies? (Y/N)
5. Do you have a policy for alternate dates for military leave? (Y/N)
6. Do you offer accommodations for disabilities per the Americans with Disabilities Act? (Y/N)
7. Do you offer lactation accommodations for postpartum women? (Y/N)

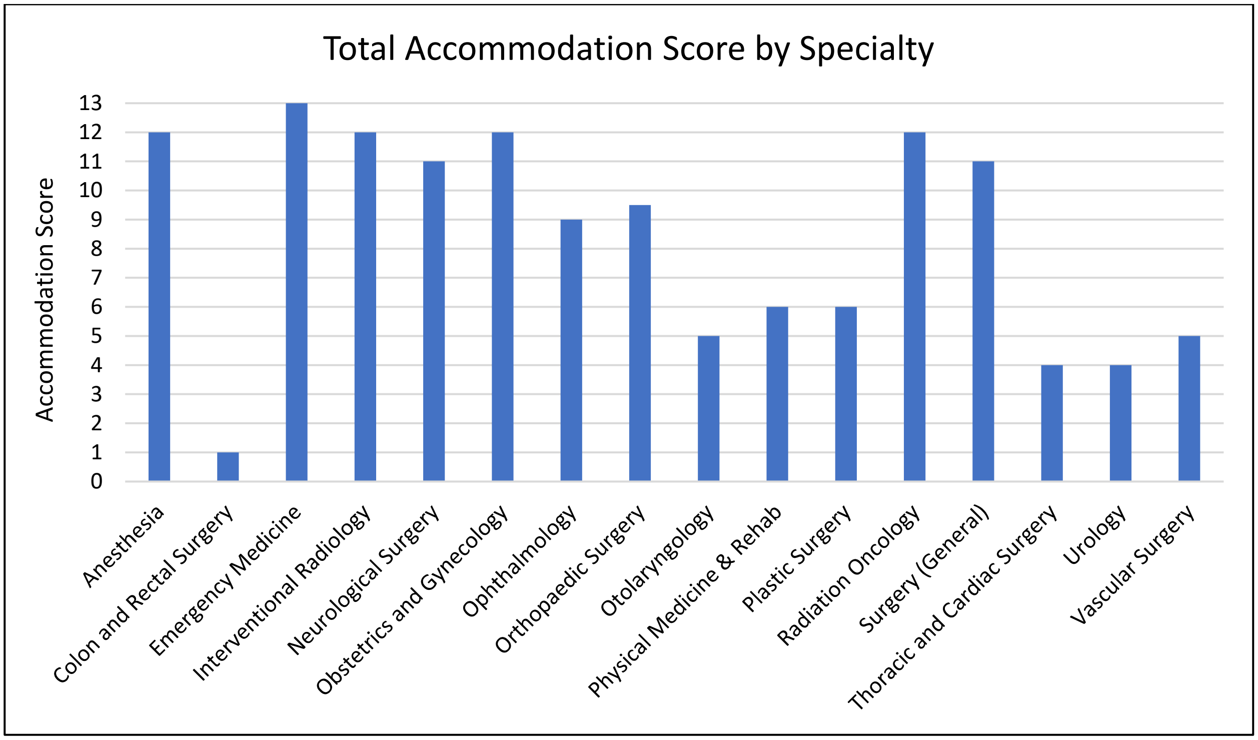

eFigure. Total Accommodation Score by Specialty

eAppendix. Policies and Practices of Oral Board Examinations

Specialty	Exam Format	No. of Exams Offered Per Year	Exam Location	Accommodations for Medical Leave/Emergency	Accommodations for Family Emergency	Accommodations for Military Leave/Deployment	Nursing/Lactation Accommodations
**Anesthesia**	In-person	6x / year	Testing Center (Raleigh) - individual rooms/stations	Yes - can submit request to change test date. No virtual alternatives offered.	Yes - can submit request to have test date changed. No virtual alternatives offered.	Yes - can submit request to have test date changed. No virtual alternatives offered.	Yes - nursing mothers can complete online form to request extra break time. Have a private room at the testing center that nursing mothers can use.
**Colon and Rectal Surgery**	In-person	1x / year	Omni Hotel (Chicago) - closed-off suite portion of hotel rooms (not the bedroom quarters)	No accommodations made - will have to postpone exam to the following year. No virtual alternatives offered.	No accommodations made - will have to postpone exam to the following year. No virtual alternatives offered.	No accommodations made - will have to postpone exam to the following year. No virtual alternatives offered.	"Potentially" could offer accommodations. Do not have an explicit policy for lactation/nursing mothers.
**Emergency Medicine**	Virtual	3x / year	Virtual	Yes - can switch to another test date within the same year.	Yes - can switch to another test date within the same year.	Yes - can switch to another test date within the same year.	Yes - candidates can have a pump during the exam and can request additional accommodations online.
**Interventional Radiology**	Virtual	2x / year	Virtual	Yes - can postpone to next exam date	Yes - can postpone to next exam date	Yes - can postpone to next exam date	Yes - offer extended break time for nursing mothers.

**Neurological Surgery**	Virtual	2x / year	Virtual	Yes - candidate is deferred to next open exam.	Yes - candidate is deferred to next open exam.<br><br>Note - Any last minute cancellations are reviewed rigorously by the Board to make sure candidates are not cancelling due to lack of preparation.	Yes - candidates can either take the exam virtually from their deployment site or switch to next open exam	No accommodations made. Ask that candidates set aside the 3 hours for the oral exam.
**Obstetrics and Gynecology**	In-person	4x / year	Testing Center (Dallas) - individual rooms/stations	Yes - candidate will get re-assigned to a new test date within the same year. No virtual alternative offered.	Yes - if candidate has legitimate family emergency, will get re-assigned to a new test date within the same year.	Yes - candidate will get re-assigned to a new test date.	Yes - candidates can request to use a dedicated lactation room before their exam.

**Ophthalmology**	Virtual	2x / year	Virtual	In general, do not allow candidates to switch dates; candidate must cancel original exam and sign up for the next one, incurring cancellation fee.<br><br>In the event of a medical illness, a letter would have to be sent in to the Board. The letter would be reviewed by leadership and then leadership will "take the next step from there" and consider whether to waive the cancellation fee.	In general, do not allow candidates to switch dates; candidate must cancel original exam and sign up for the next one, incurring cancellation fee.<br><br>Candidates may submit a letter to the Board documenting the family emergency. The letter would be reviewed by leadership and then leadership will "take the next step from there".	In general, do not allow candidates to switch dates; candidate must cancel original exam and sign up for the next one, incurring cancellation fee.<br><br>Candidates may submit a letter to the Board documenting their military leave. The letter would be reviewed by leadership.	Yes - will add 60-minutes extra break time for nursing mothers

© 2024 Rowe DG et al. *JAMA Network Open.*

**Orthopaedic Surgery**	In-person	2x / year	Site 1: Palmer House Hotel (Chicago) - exams conducted in booths created in the exhibit space on the 3rd/4th floors of the hotel<br><br>Site 2: Testing Center (Raleigh) - individual rooms/stations	Yes - can switch to next exam date. No virtual alternatives offered.	Yes - can switch to next exam date. No virtual alternatives offered.	Yes - can switch to next exam date. No virtual alternatives offered.	Site 1: no lactation rooms available or additional break time offered.<br><br>Site 2: lactation rooms available for nursing mothers. No additional break time offered.
**Otolaryngology**	Virtual	1x / year	Virtual	No accommodations made - will have to postpone exam to the following year.	No accommodations made - will have to postpone exam to the following year.	No accommodations made - will have to postpone exam to the following year.<br>However, the Board will "try to schedule around military leave if candidates know in advance."	No accommodations made. Candidates have a break between their exams sessions that they may use to pump
**Physical Medicine & Rehab**	Virtual	1x / year	Virtual	No accommodations made - will have to postpone exam to the following year.	No accommodations made - will have to postpone exam to the following year.	Unclear policy regarding military deployment - "potentially could work around this."	Yes - nursing mothers can request extended break time.

**Plastic Surgery**	In-person	1x / year	Pointe Hilton (Phoenix) - suite portion of hotel rooms	Candidate can opt to take the exam virtually or can postpone by a year.	Candidate can opt to take the exam virtually or can postpone by a year.	Candidate can opt to take the exam virtually or can postpone by a year.	No accommodations made. Noted that the exams are only 45 minutes so candidates should not need extra time to pump.
**Radiation Oncology**	Virtual	2x / year	Virtual	Yes - can switch to next exam date.	Yes - can switch to next exam date.	Yes - can switch to next exam date.	Yes - nursing mothers are offered extended break time.
**Surgery (General)**	Virtual	2x / year	Virtual	Yes - can switch to next available exam date.	Yes - can switch to next available exam date.	Yes - can switch to next available exam date.	No accommodations made. Noted that the exams are short (1.5-2 hours long) so candidates should not need extra time to pump.
**Thoracic and Cardiac Surgery**	In-person	1x / year	Testing Center (Dallas) - individual rooms/stations	No accommodations made - will have to postpone exam to the following year. No virtual alternatives offered.	No accommodations made - will have to postpone exam to the following year. No virtual alternatives offered.	No accommodations made - will have to postpone exam to the following year. No virtual alternatives offered.	Yes - candidates can request to use a dedicated lactation room.
**Urology**	In-person	1x / year	Testing Center (Raleigh) - individual rooms/stations	No accommodations made - will have to postpone exam to the following year. No virtual alternatives offered. Can extend window of admissibility for excused absence.	No accommodations made - will have to postpone exam to the following year. No virtual alternatives offered. Can extend window of admissibility for excused absence.	No accommodations made - will have to postpone exam to the following year. No virtual alternatives offered. Can extend window of admissibility for excused absence.	Yes - candidates can request to use a dedicated lactation room and will get extra break time if needed.

**Vascular Surgery**	Virtual	1x / year	Virtual	No accommodations made - will have to postpone exam to the following year.	No accommodations made - will have to postpone exam to the following year.	No accommodations made - will have to postpone exam to the following year.	"Typically we haven't had to accommodate nursing mothers because the exam is only an hour and a half. But individuals can request any accommodation through the website."
