# Supplementary material for: Variation in Oral Board Examination Accommodations Among Specialties
Source: JAMA Netw Open. 2024 May 7;7(5):e2410127. doi: 10.1001/jamanetworkopen.2024.10127 (PMC11077388; doi:10.1001/jamanetworkopen.2024.10127)
Supplement: Supplement 2. — Data Sharing Statement [file jamanetwopen-e2410127-s002.pdf]

Data Sharing Statement

Rowe. Variation in Oral Board Examination Accommodations Among Specialties. *JAMA Netw Open*. Published May 07, 2024. doi:10.1001/jamanetworkopen.2024.10127

Data
Data available: Yes
Data types: Data (not involving human participants)
How to access data: melissa.erickson@duke.edu
When available: With publication

Supporting Documents
Document types: None

Additional Information
Who can access the data: researchers whose proposed use of the data has been approved
Types of analyses: for approved purposes
Mechanisms of data availability: Shared via excel after approval of proposal by principle investigator
